# Cytochrome P450 enzymes in the black-spotted frog (*Pelophylax nigromaculatu*s): molecular characterization and upregulation of expression by sulfamethoxazole

**DOI:** 10.3389/fphys.2024.1412943

**Published:** 2024-05-09

**Authors:** Zhiqun Liu, Chaoli Shi, Bingyi Wang, Xiaofang Zhang, Jiafeng Ding, Panpan Gao, Xia Yuan, Zhiquan Liu, Hangjun Zhang

**Affiliations:** ^1^ Hangzhou Normal University, Hangzhou, China; ^2^ Zhejiang Provincial Key Laboratory of Urban Wetlands and Regional Change, Hangzhou, China; ^3^ State Environmental Protection Key Laboratory of Environmental Health Impact Assessment of Emerging Contaminants, Shanghai Academy of Environment Sciences, Shanghai, China

**Keywords:** *Pelophylax nigromaculatus*, sulfamethoxazole, cytochrome P450, expression analysis, molecular docking

## Abstract

Cytochrome P450 (CYP) enzymes are crucial for the detoxification of xenobiotics, cellular metabolism, and homeostasis. This study investigated the molecular characterization of CYP enzymes in the black-spotted frog, *Pelophylax nigromaculatus*, and examined the regulation of CYP expression in response to chronic exposure to the antibiotic sulfamethoxazole (SMX) at various environmental concentrations (0, 1, 10, and 100 μg/L). The full-length cDNA of Pn-CYP26B1 was identified. The sequence included open reading frames of 1,536 bp, encoding proteins comprising 511 amino acids. The signature motif, FxxGxxxCxG, was highly conserved when compared with a number of selected animal species. SMX significantly upregulated the expression of the protein CYP26B1 in frog livers at concentrations of 1 and 10 μg/L. SMX showed an affinity for CYP26B1 of −7.6 kcal/mol, indicating a potential mechanism for SMX detoxification or adaptation of the frog. These findings contributed to our understanding of the environmental impact of antibiotics on amphibian species and underscored the importance of CYP enzymes in maintaining biochemical homeostasis under exposure to xenobiotic stress.

## 1 Introduction

Antibiotics have emerged as a new category of pollutant, eliciting widespread public concern due to their potential ecological and biological health threats (Kümmerer, 2009). Antibiotics, including β-lactams, macrolides, quinolones, tetracyclines, sulfonamides, aminoglycosides, and chloramphenicol, are crucial in the treatment of infectious diseases in aquaculture and livestock (Shao et al., 2021). Antibiotics are now widely distributed pollutants in aquatic environments, including all types of surface water. Rivers, lakes, and coastal zones now constitute a principal reservoir and conduit for antibiotic accumulation and spread (Liu et al., 2018). One of most commonly used antibiotics, sulfamethoxazole (SMX) has been frequently detected in various aquatic environments, including drinking water, groundwater, surface water, and wastewater treatment plant effluents, in concentrations varying from 2.9 to 216 ng/L (Huang et al., 2020; Zainab et al., 2020; Cui et al., 2021). In particular, SMX concentrations in wastewater effluents range from 200 to 2,000 ng/L (Christou et al., 2017). The bioaccumulation of SMX through the food chain may adversely affect aquatic life (Yang et al., 2024). As it becomes more broadly disseminated and bacterial antibiotic resistance becomes more widespread, SMX constitutes a burgeoning environmental pollution issue that poses risks to ecosystems and human health.

Amphibians, many species of which primarily inhabit agricultural areas, serve as indicators for environmental and ecological health and have experienced significant declines in population size and species diversity in recent decades (Jiang et al., 2016). Their vulnerability to antibiotics, such as SMX, stems from their lack of a protective eggshell, highly permeable skin, and the aquatic environments in which their embryos and larvae develop (Alford et al., 2001). Studies have shown that SMX can induce hepatocellular damage in amphibians, adversely affecting their growth, development, and behavior (Rutkoski et al., 2022). The black-spotted frog, *Pelophylax nigromaculatus* is characterized by its highly permeable skin and sensitivity to environmental pollutants, and has suffered a reduction in its global distribution (Lin et al., 2022). Increasing evidence suggests that environmental pollutants are contributing to declining frog population worldwide, prompting the selection of this species for this study of SMX toxicity (Wang et al., 2019).

The cytochrome P450 (CYP) enzymes are a group of membrane-bound hemoproteins found in nearly all living organisms, and play a vital role in synthesizing various endogenous compounds (Baune et al., 1999; Pikuleva and Cartier, 2021; Pang et al., 2022). As major phase I metabolizing enzymes, CYP enzymes biotransform xenobiotics, such as antibiotics, within the body (Ha-Duong et al., 2001; Lim et al., 2024). While some studies indicate that antibiotics can influence CYP enzyme activity in aquatic organisms, there is little comprehensive research on the transcriptional profiles of specific classes of CYP (Samrani et al., 2023). In contrast, the influence of other pollutants, such as polycyclic aromatic hydrocarbons, crude oil and nanoplastics, on the gene expression patterns of CYP enzymes has been studied more, suggesting a need for a more focused examination of the molecular mechanisms of antibiotics (Han et al., 2015; Lie et al., 2019; Wu et al., 2019). The cytochrome P450 family 26 (CYP26) enzymes are crucial for retinoic acid (RA) metabolism and homeostasis in humans, mammals, and other chordates, efficiently metabolizing all-trans-retinoic acid and its isomers, as well as primary metabolites (White et al., 2000; Maden, 2002). The CYP26 enzyme is regulated by both inflammatory cytokines and endogenous processes, exhibiting tissue and cell-type-specific expression patterns in animal models (Lampen et al., 2001; Rydeen and Waxman, 2014). Notably, the loss of CYP26B1 in mice causes alveolar inflation failure and alveolar type 1 cell reduction (Daniel et al., 2020). In zebrafish, tight control of CYP26B1 activity and RA levels is critical for skeletogenesis (Spoorendonk et al., 2008). However, data linking emerging pollutants to altered CYP expression or activity is scarce, and the impact of environmental pollutants, particularly antibiotics, on CYP activity is not yet fully understood.

To address this gap, we identified the full-length cDNA of the CYP26B1 in *P. nigromaculatus*, and examined its transcriptional response to various environmental concentrations of SMX to elucidate the regulation of CYP26B1 under SMX stress. This study aimed to determine whether chronic exposure to SMX could activate the phase I detoxification system in *P. nigromaculatus*. This study enhanced our understanding of the biochemistry of CYP in frogs, especially *P. nigromaculatus*, and the gene expression patterns in response to environmental contaminants, especially antibiotics. It should enable further investigation into the molecular mechanisms of the effects of antibiotic pollution on amphibians.

## 2 Materials and methods

### 2.1 Chemicals

Sigma-Aldrich (Shanghai, China) and Yeasen Biotechnology (Shanghai, China) provided analytical standard SMX (CAS No. 763-46–6, Product Number: 31,737, purity ≥98.0%) and dimethyl sulfoxide (DMSO) (CAS No. 67-68–5, purity >99.9%), respectively. All of the other reagents used were of chromatographic or analytical grade.

### 2.2 Culture of frogs

Healthy *P. nigromaculatus* for our study were obtained from the ChangXing Agriculture Development Co., Ltd (Huzhou, China). To ensure their wellbeing and acclimatization, the frogs were housed in laboratory conditions with a natural light-dark cycle in distilled water tanks at a temperature of 20°C ± 1°C. The water quality parameters were carefully monitored, with dissolved oxygen content maintained at 7 ± 1 mg/L and pH at 6.5 ± 0.5. During a 2-week acclimatization period, the water in the tanks was completely refreshed every 24 h to ensure optimal dissolved oxygen levels.

### 2.3 Experimental design

After the acclimatization period, healthy male frogs, weighing approximately 30 g, were used for the exposure experiment. Males were selected based on the field observation that wild males may have higher levels of pollutants in their bodies than females (Cui et al., 2018). Considering the known range of environmental SMX concentrations and drawing on previous studies into the mechanism of SMX toxicity (Bielen et al., 2017; Xiong et al., 2019), the frogs were exposed to several SMX concentrations for a period of 21 days. Each experimental group consisted of three replicate aquaria, each measuring 30 cm × 30 cm × 60 cm. Twenty frogs were placed into each aquarium and exposed to either a test SMX solution or a 0.01% DMSO solution, which served as the control. The exposure solutions and control solutions were carefully prepared to maintain a constant temperature of 20°C ± 1°C, pH of 6.5 ± 0.5, and dissolved oxygen content of 7 ± 1 mg/L. The groups comprised a control group treated with 0.01% DMSO and three experimental groups exposed to concentrations of 1, 10, and 100 μg/L of SMX. After 21 days exposure, the frogs were humanely euthanized and their bodies were carefully dissected. Liver samples were collected from the dissected frogs and stored at −80°C for further analysis. All of the procedures strictly adhered to the guidelines established by the Association of Laboratory Animal Sciences, to ensure the ethical treatment of the frogs throughout the study.

### 2.4 PCR RNA isolation and cDNA synthesis

A rigorous methodology was employed to isolate total RNA from the liver samples (n = 3 replicates), using TRIzol reagent (Beijing ComWin Biotech Co., Ltd., Beijing, China). The completeness and purity of the isolated RNA was tested using electrophoresis on a 1.2% agarose gel in a Nanodrop spectrophotometer (Thermo Fisher Scientific, Waltham, MA, United States). Contaminated genomic DNA was eliminated and Hifair^®^ III 1st Strand cDNA Synthesis SuperMix for qPCR (gDNA digester plus) kits (Yeasen Biotechnology Co., Ltd., Shanghai, China) were used for the reverse transcription of RNA.

### 2.5 Bioinformatics sequence analysis

Amino acid sequences and open reading frames (ORFs) were predicted using ORFFinder (https://www.ncbi.nlm.nih.gov/orffinder/). Sequence homology was analyzed using BLASTP (https://blast.ncbi.nlm.nih.gov/). The prediction of signal peptides and amino acid functional domains was conducted using SignalP v.6.0 (https://services.healthtech.dtu.dk/services/SignalP-6.0/) and ExPASy-PROSITE (http://prosite.expasy.org/), respectively. Secondary structure analysis of proteins was performed using PSIPRED Workbench (http://bioinf.cs.ucl.ac.uk/psipred/). Protein transmembrane helical structures were predicted using TMHMM (https://services.healthtech.dtu.dk/services/TMHMM-2.0/). Pairwise and multiple sequence alignments were performed using ClustalX and ESPript (http://espript.ibcp.fr/ESPript/ESPript/). Phylogenetic analysis was conducted by constructing a neighbor-joining tree based on the alignments using the MEGA 11.0 software (https://www.megasoftware.net/).

### 2.6 Quantitative real-time polymerase chain reaction (qRT-PCR) analyses

QRT-PCR analysis of the CYP in *P. nigromaculatus* was performed after 21 days of exposure to three different SMX concentrations (1, 10, 100 μg/L) and a DMSO control (Supplementary Figure S1). RNA extraction and cDNA synthesis were performed as described in Section 2.4. QRT-PCR was conducted using Hieff^®^ qPCR SYBR^®^ Green Master Mix (No Rox) kits (Yeasen Biotechnology Co., Ltd., Shanghai, China) on a CFX 96 Touch Real-Time PCR Detection System (Bio-Rad, Feldkirchen, Germany), following the protocol: initial denaturation at 95°C for 5 min; followed by 40 cycles at 95°C for 10 s, 60°C for 20 s, and 72°C for 20 s. Specific primers for target genes were designed using the Primer Premier v.6.0 software (https://primer-premier.software.informer.com/6.0/). The primer sequences used for the qRT-PCR are described in Supplementary Table S1. All primer pairs produced a single dissociation peak in every reaction, confirming specificity, and no amplification was observed in template-absent reactions. Due to its stable expression across all exposure groups, actin was selected as the reference gene for the transcriptional assay. The relative mRNA levels of the target genes were determined using the 2^−ΔΔCT^ method (Livak and Schmittgen, 2001).

### 2.7 Molecular docking

ORFFinder was used to translate the gene sequence into amino acid sequences for protein encoding, followed by alignment using BLAST search (BLASTP). The 3D structures were constructed using a homology modeling approach using the CYP26B1 template (PDB entry 2VE3) in the Alignment Mode of SWISS-MODEL (https://swissmodel.expasy.org/). The 3-D structures of SMX were acquired from ZINC (http://zinc.docking.org/) using CAS numbers. Prior to molecular docking, PlayMolecule (https://www.playmolecule.com/) was used to predict ligand binding pockets, and AutoDock (https://autodock.scripps.edu/) Tools v.1.5.7 to prepare the ligands and receptors. Protein modeling involved removing water molecules and extraneous ligands, adding hydrogen atoms, and applying Kollman charges. The grid box, central to the core site (43.61, −1.36, 10.59) of CYP26B1, was established with a coverage of 60 × 60 × 60 Å^3^. AutoDock Vina was used to conduct the molecular docking, with 10 independent runs per docking and the binding mode selected for the lowest energy for analysis. The results displaying the lowest docked energy were examined visually using the Discovery Studio Visualizer 2021 Client (San Diego, CA, United States).

### 2.8 Data analysis

Data were presented as means ± standard error. Statistical variances between the control and treatment groups were determined using one-way analysis of variance (ANOVA) followed by Tukey’s test, with significance set at *p* < 0.05. Normality and homogeneity of the data were validated before the ANOVA analysis using the Shapiro-Wilk’s test and Levene’s test. For skewed data distributions, the significant Kruskal–Wallis test was employed, followed by the Dunn-Bonferroni *post hoc* method. All statistical analyses were conducted using SPSS Statistics v.27.0 (IBM Corporation, Somers, NY, United States).

## 3 Results and discussion

### 3.1 Molecular characterization

**FIGURE 1:**
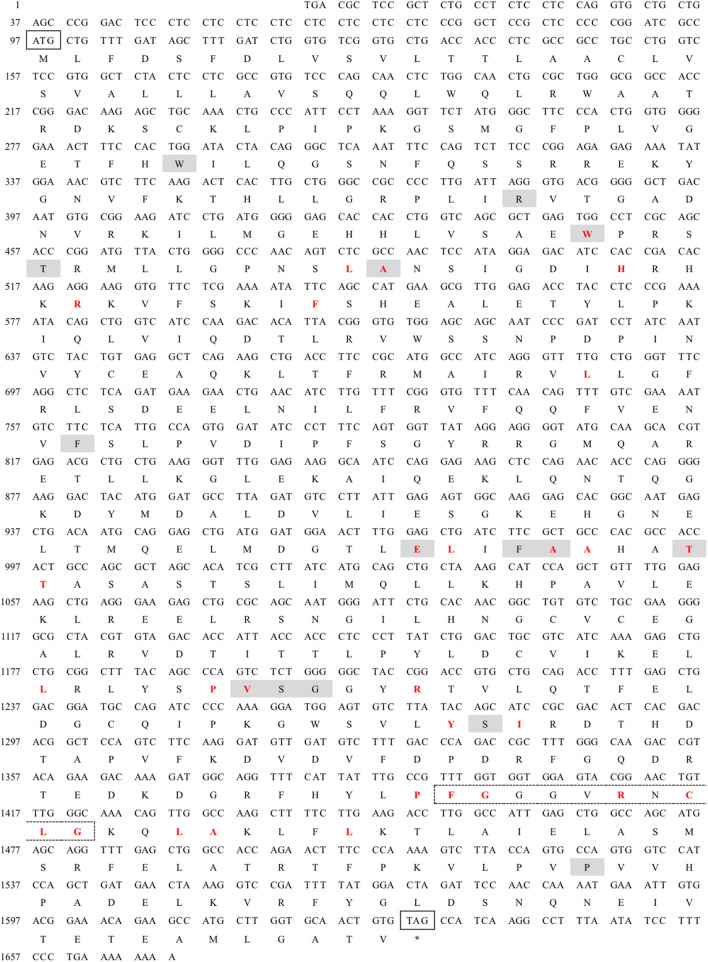
Nucleotide sequence and deduced amino acid sequences of *Pelophylax nigromaculatus* CYP26B1. The start (ATG) and stop (TAG) codons are indicated by solid boxes, and the stop codon is marked with an asterisk. The heme binding site is highlighted in red, while the chemical substrate binding pockets are shaded. The heme-iron ligand signature sequence (FXXGXRXCXG) is identified by a dashed box.

**FIGURE 2:**
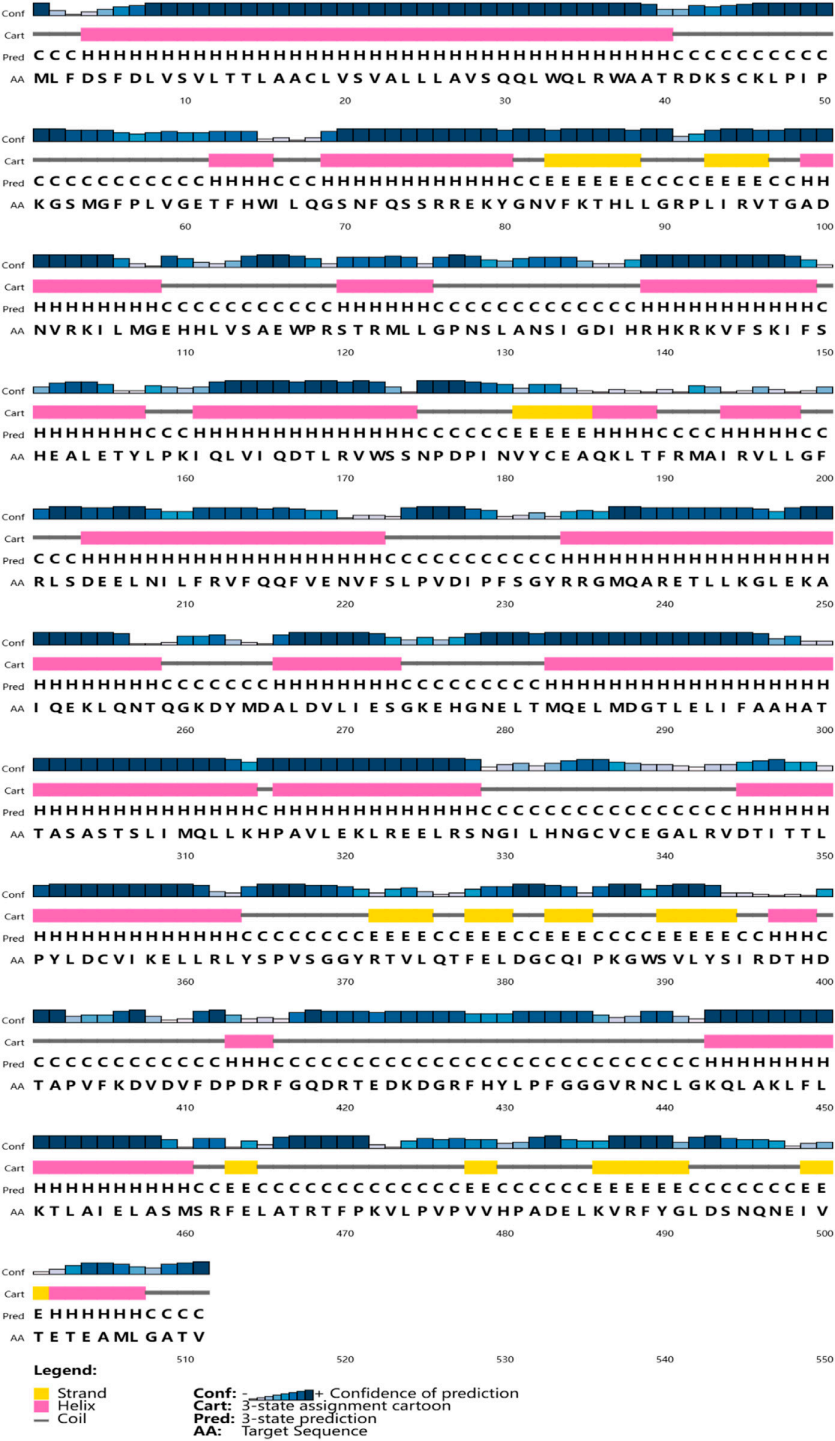
The secondary structures of Pn-CYP26B1. Strands and helixes are indicated by yellow and pink boxes, respectively.

The full-length cDNA of CYP26B1 from *P. nigromaculatus* was shown to be 1,669 bp in length, using our prior transcriptome sequencing data (Figure 1). To the best of our knowledge, this is the first reported instance of a full-length CPY gene cDNA in *P. nigromaculatus*. The sequence included ORFs of 1,536 bp, encoding proteins comprising 511 amino acids. Pn-CYP26B1 possessed a 96 bp UTR at the 5′-end and a 37 bp UTR at the 3′-end. The molecular mass and theoretical isoelectric point of Pn-CYP26B1 were determined as 57.64 kDa and 7.65, respectively (Table 1). Pn-CYP26B1 showed several characteristics common to the P450 superfamily, including function-critical sequence motifs that are highly conserved throughout evolution and pivotal for heme binding within the CYP26B1 sequences (Zhang et al., 2016). Following protein functional domain analysis, we identified a transmembrane helix region spanning positions 7–29 (Supplementary Figure S2), a conserved functional domain for CPY spanning 50–465 (PFAM00067), and a putative cysteine heme-iron ligand signature spanning 433–442 (FGLGKRSCIG) (PS00086). The estimated percentages of coil, helix, and strand structures were 41.49%, 50.10%, and 8.41%, respectively (Figure 2).

**TABLE 1:** Details of the full-length cDNA sequences of GSTα and CYP26B1 in the frog, *Pelophylax nigromaculatu*s.

	Pn-CYP26B1
Full Length (bp)	1,669
5′-UTR (bp)	96
3′-UTR (bp)	37
ORF (bp)	1,536
Number of amino acids	511
Protein isoelectric point	7.65
Molecular weight	57,637.72

### 3.2 Multiple sequence alignment

**FIGURE 3:**
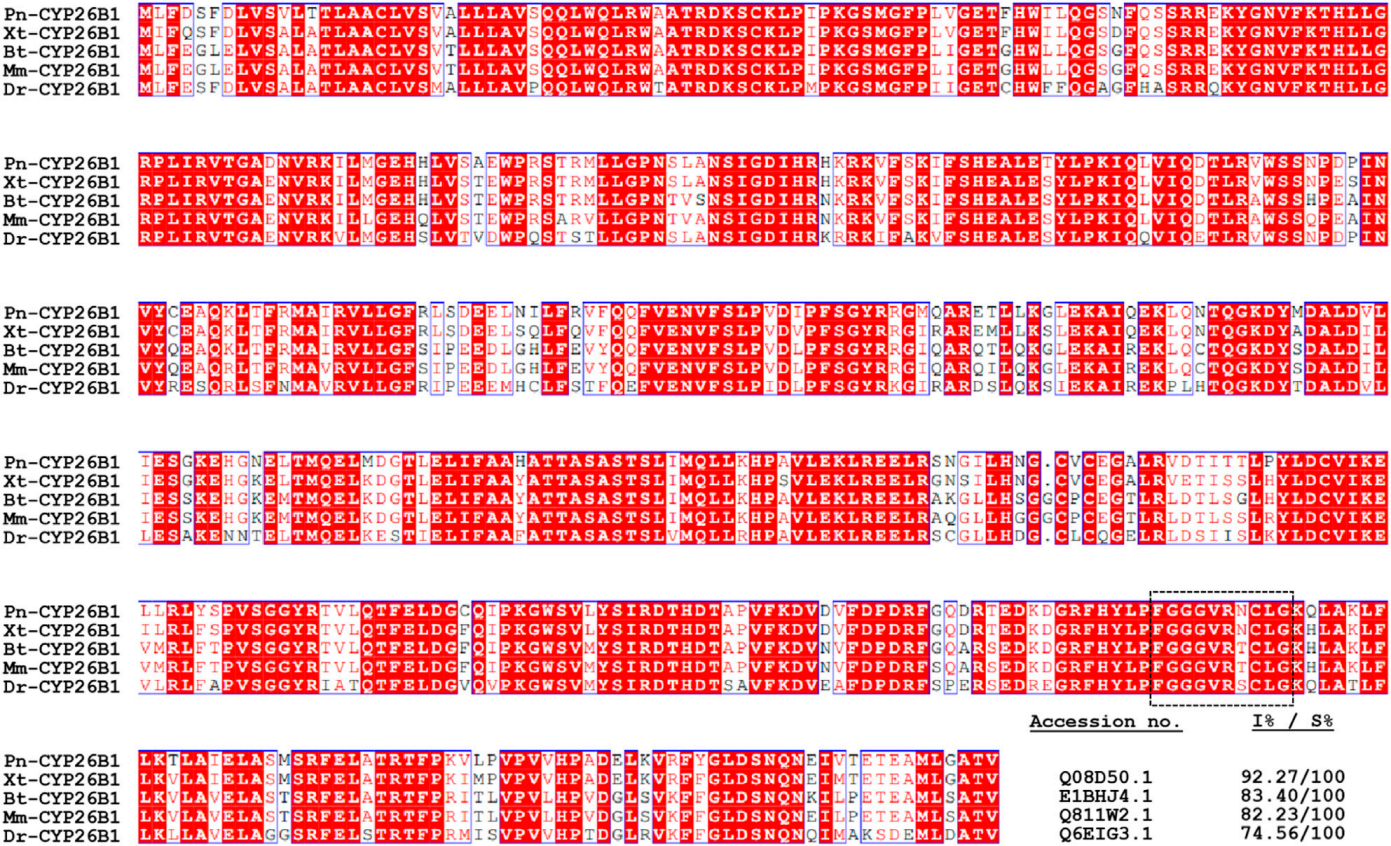
Multiple amino acid sequence alignment of *Pelophylax nigromaculatus* CYP26B1 with known homologs from other species. Completely (100%) conserved residues are shown in white text against a red background. Residues shown in red signify strong conservation. The heme-binding motif, FXXGXRXCXG, is outlined using dashed lines. The GenBank accession numbers and percentages of identity (I%) and similarity (S%) for the aligned sequences are provided in the lower right-hand section of the figure.

A multiple alignment analysis comparing Pn-CYP26B1 with the CYPs of other species revealed a high degree of homology. Pn-CYP26B1 exhibited 100% identity with sequences from *Bos taurus*, *Danio rerio*, *Mus musculus*, and *Xenopus tropicalis*. Among these animal species, the signature motif, FxxGxxxCxG, was highly conserved (Figure 3). A crucial feature of CYP enzymes is their heme-centered active site, featuring a Cys axial ligand. This identifies them as heme-thiolate proteins, primarily active as monooxygenases despite their conventional classification as cytochromes (Dermauw et al., 2020). Based on the systematic organization of the Cytochrome P450 Nomenclature Committee, the deduced amino acid sequence of Pn-CYP26B1 belonged to the CYP family 26, subfamily B, polypeptide 1.

### 3.3 Phylogenetic characterization

**FIGURE 4:**
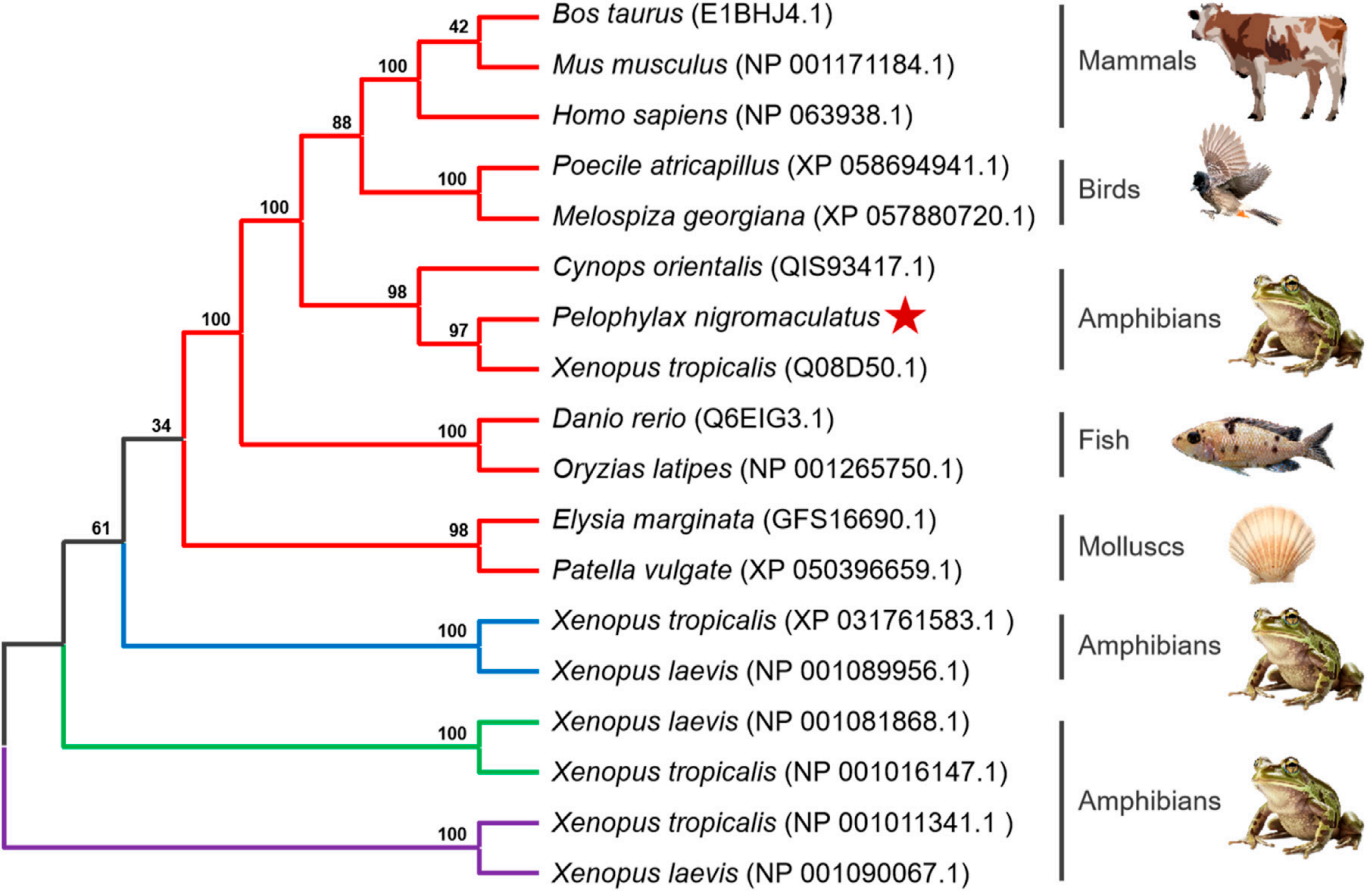
Un-rooted phylogenetic tree portraying the relationship between *Pelophylax nigromaculatus* CYP26 with *CYP* genes from other organisms. Each color represents a different class of *CYP* genes: red for CYP26B1; blue for CYP26C1; green for CYP26A1; and purple for CYP27C1. This tree, derived from the alignment of full-length amino acid sequences, was generated using the Neighbor-Joining (NJ) method in ClustalW and MEGA 11, and was bootstrapped 1,000 times.

To determine the evolutionary position of Pn-CYP26B1, the Neighbor-Joining method (Saitou and Nei, 1987) was used to construct an unrooted phylogenetic tree. Our analysis included the CPY450 family members from both invertebrate and vertebrate species. The resulting phylogenetic tree not only clearly distinguished between invertebrate and vertebrate CYPs but also revealed four distinct groups, the classes CYP26B1, CYP26A1, CYP26C1, and CYP27C1, which were clearly separated (Figure 4). Three CPY26 enzymes, namely, CYP26A1, CYP26B1, and CYP26C1, have been previously identified in vertebrates (White et al., 1996; Nelson, 1999; Gu et al., 2005). CYP26 and CYP27 were classified into distinct CYP450 families, consistent with the branching results of our phylogenetic tree. CYPs belonging to the same class formed tight clusters, demonstrating their close evolutionary relationships. The protein encoded by Pn-CYP26B1 grouped first, with its ortholog in *X. tropicalis*, and subsequently clustered with other vertebrates. Although molluscs and vertebrates having the same CYP26B1 were grouped together, their evolutionary affinity was low. CYP26B1 did not cluster with the amphibian proteins encoded by CYP26C1, CYP26A1, and CYP27C1, highlighting its distinct evolutionary trajectory.

### 3.4 Tertiary structure

**FIGURE 5:**
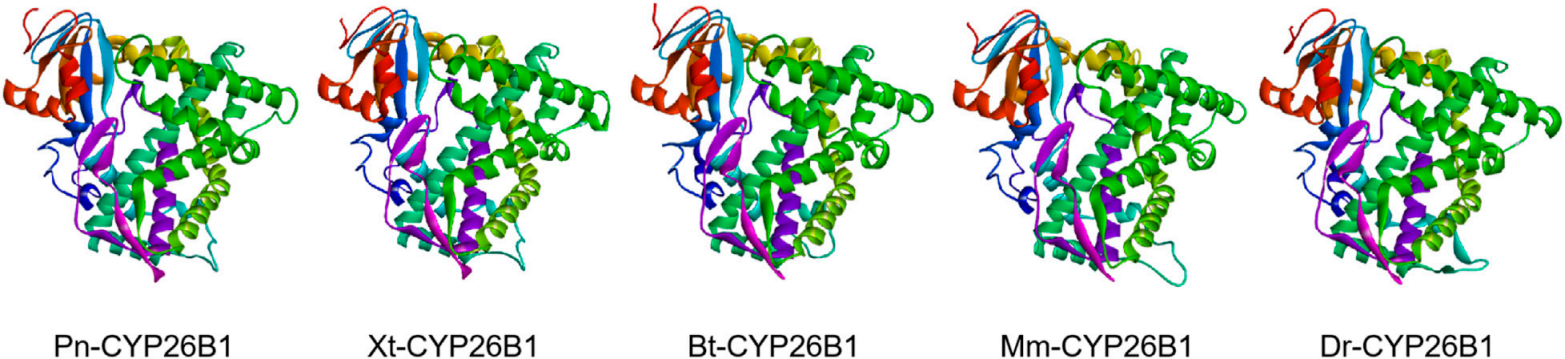
The predicted spatial structures of Pn-CYP26B1, Xt-CYP26B1, Bt-CYP26B1, Mm-CYP26B1, and Dr-CYP26B1. These structures were predicted using the SWISS-MODEL program, based on the crystal structure of *Synechocystis* sp. *PCC 6803* CYP (PDB entry 2VE3) as a reference for CYP26B1. Pn: *Pelophylax nigromaculatus*. Xt: *Xenopus tropicalis*. Bt: *Bos taurus*. Mm: *Mus musculus*. Dr: *Danio rerio*.

Based on the template of the crystal structure of *Synechocystis* sp. PCC 6803 CYP (PDB entry 2VE3), the potential tertiary structures of CYP26B1 from *B. taurus*, *D. rerio*, *M. musculus*, *P. nigromaculatus*, and *X. tropicalis* were established using the SWISS-MODEL prediction algorithm (Figure 5). The similarity between the template and the Pn-CYP26B1, Xt-CYP26B1, Bt-CYP26B1, Mm-CYP26B1, and Dr-CYP26B1 sequences ranged from 34.87% to 37.04%. The Global Model Quality Estimations were >0.68 (Table 2), indicating the high quality of these results, and the Qualitative Model Energy Analyses were > −1.64 (Table 2).

**TABLE 2:** Sequence identity, GMQE, and QMEAN for GSTα and CYP26B1 in different organisms.

(B)	Gene name (B)	Seq identity (%)	GMQE	Qmean
CYP261	Pn-CYP261	35.01	0.69	−1.44
	Xt-CYP261	36.49	0.69	−1.38
	Bt-CYP261	34.87	0.68	−1.53
	Mm-CYP261	35.42	0.68	−1.35
	Dr-CYP261	37.04	0.68	−1.64

Pn, *Pelophylax nigromaculatus*; Xt, *Xenopus tropicalis*; Bt, *Bos taurus*; Mm, *Mus musculus*; Dr, *Danio rerio*.

### 3.5 Binding and activation characteristics of SMX with CYP26B1

#### 3.5.1 Aberrant expressions of the CYP26B1 proteins

**FIGURE 6:**
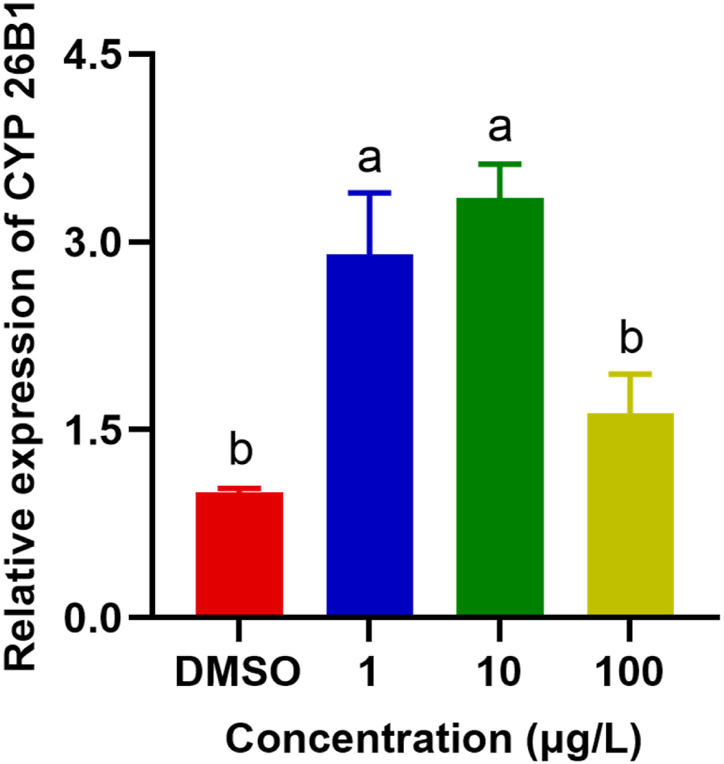
Effects of SMX exposure at different concentrations on the expression of CYP26B1 in the livers of *Pelophylax nigromaculatus*. Values are given as mean ± SE (n = 3). Significant differences are denoted by differing letters (*p* < 0.05).

CYP enzymes are predominantly found in the microsomal fraction of livers, where they play a vital role in bile acid biosynthesis and the metabolism of xenobiotics (Zanger and Schwab, 2013; Manikandan and Nagini, 2018). In addition, they contribute significantly to the homeostasis of steroid hormones, being located in the inner mitochondrial membrane within the steroidogenic tissues (Estabrook et al., 1963). They are also crucial in the metabolism of vitamins, unsaturated fatty acids, and cholesterol (Lane et al., 1999; Omura, 1999). SMX caused significant upregulation of the expression of CYP26B1 in *P. nigromaculatus* livers at concentrations of 1 and 10 μg/L (*p* < 0.05) (Figure 6), but no significant upregulation at 100 μg/L, compared with the control (*p* > 0.05). They are pivotal in xenobiotic metabolism within hepatocytes and central to biotransformation processes. Beyond hydroxylation, CYP enzymes facilitate diverse biotransformation reactions, e.g., dehalogenation, dehydrogenation, oxygenation, epoxidation, and dealkylation. Phase I metabolism also involves non-CYP mediated pathways through flavin-containing monooxygenases, amine oxidases, alcohol dehydrogenases, esterases, and peroxidases (Esteves et al., 2021). As with our findings, previously observed expression patterns of the four *CYP* genes in *Daphnia pulex* all exhibited elevation at low nanoplastic treatment doses, and reduction at higher doses without significant differences compared to the control (Wu et al., 2019). Elevated concentrations of SMX probably induced increased toxicity and a shift in metabolic homeostasis, potentially leading to the downregulation of numerous metabolism-related genes (Zhang et al., 2020; Huang et al., 2021).

#### 3.5.2 Binding of SMX to the CYP26B1 proteins

**FIGURE 7:**
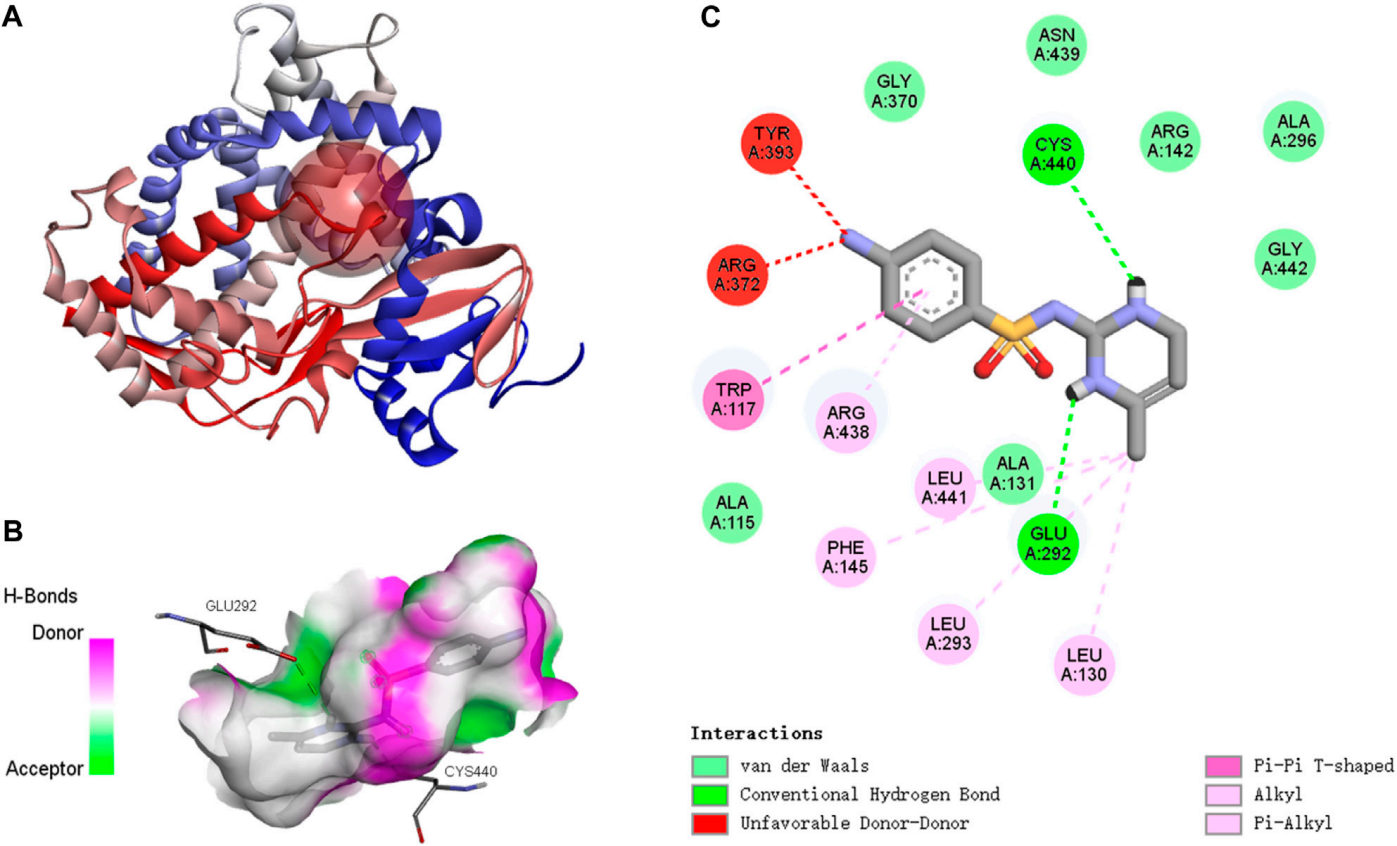
Molecular docking results of SMX with *Pelophylax nigromaculatus* CYP26B1. **(A)** The active binding sites. **(B)** The docking mode. **(C)** The hydrogen bond interactions.

The active binding sites of SMX to the *P. nigromaculatus* CYP26B1 proteins are shown in Figure 7A. The docking modes of SMX onto the CYP26B1 proteins are shown in Figure 7B and the hydrogen bond interactions are shown in Figure 7C. SMX showed an affinity of −7.6 kcal/mol for CYP26B1. Hydrogen bonding plays a major role in ligand binding to target receptors. The affinity of a ligand for the CYP26B1 protein can influence its activity and mediate its effects. In this study, a strong affinity between SMX and the CYP26B1 proteins was observed. CYP26 enzymes are essential for maintaining RA homeostasis by regulating its availability for receptor binding and signaling (Roberts, 2020). RA signaling plays a vital role in a myriad of biological functions across organisms, including modulation of immune responses (Oliveira et al., 2018). It also contributes to the metabolism of lipids and fatty acids, energy metabolism, adipocyte differentiation and remodeling, and the regulation of postnatal skeletal growth and homeostasis (Williams et al., 2009). The primary role of CYP26 is to degrade endogenous all-trans RA, which is found in high concentrations in tissues (Perlmann, 2002). This acid efficiently binds to retinoic acid receptors (RARs) and represents the primary biologically active retinoid *in vivo* (Horton and Maden, 1995). Retinoids, which are crucial in cell signaling, bind to two classes of retinoid receptors, RARs and retinoid X receptors. These ligand-regulated transcription factors are essential for development and physiology (Carvalho et al., 2017). The importance of retinoids is well-documented, with significant developmental abnormalities arising from either retinoid deficiency or excess (Emoto et al., 2005). Embryos from CYP26-deficient mice display defects akin to those resulting from all-trans-retinoic acid-induced teratogenicity (Uehara et al., 2009). It is hypothesised that activation of CYP26, which functions in retinoic acid hydroxylation, increases the metabolism and excretion rate of all-trans retinoic acid. This, in turn, raises the demand for all-trans retinoic acid precursors such as retinol and retinal, resulting in the depletion of retinoid stores, as observed in Atlantic salmon (*Salmo salar*) following exposure to benzo(a)pyrene (Beníšek et al., 2011; Berntssen et al., 2016). Abnormal expression of CYP26B1, induced by SMX, may disrupt RA homeostasis, potentially causing harm to organisms.

## 4 Conclusion

Our research provided novel insights into the molecular and functional dynamics of CYP26B1, a critical enzyme in the detoxification pathway, in *P. nigromaculatus* exposed to SMX. The study confirmed that SMX exposure significantly upregulated the expression of CYP26B1, suggesting its involvement in mitigating antibiotic-induced toxicity. This adaptive response underscored the role of CYP enzymes in the environmental resilience and health of amphibians, offering a molecular basis for further investigations into the impacts of emerging pollutants on aquatic wildlife. Furthermore, our findings emphasized the need for environmentally sustainable practices in antibiotic usage to mitigate its pervasive effects on non-target organisms and ecosystems.

## Data Availability

The datasets presented in this study can be found in online repositories. The names of the repository/repositories and accession number(s) can be found in the article/Supplementary Material.

## References

[B1] Alford R. A. Dixon P. M. Pechmann J. H. K. (2001). Ecology. Global amphibian population declines. Nature 412 (6846), 499–500. 10.1038/35087658 11484041

[B2] Baune B. Flinois J. P. Furlan V. Gimenez F. Taburet A. M. Becquemont L. (1999). Halofantrine metabolism in microsomes in man: major role of CYP 3A4 and CYP 3A5. J. Pharm. Pharmacol. 51 (4), 419–426. 10.1211/0022357991772628 10385214

[B3] Beníšek M. Kubincová P. Bláha L. Hilscherová K. (2011). The effects of PAHs and N-PAHs on retinoid signaling and Oct-4 expression *in vitro* . Toxicol. Lett. 200 (3), 169–175. 10.1016/j.toxlet.2010.11.011 21111795

[B4] Berntssen M. H. Ørnsrud R. Rasinger J. Søfteland L. Lock E. J. Kolås K. (2016). Dietary vitamin A supplementation ameliorates the effects of poly-aromatic hydrocarbons in Atlantic salmon (*Salmo salar*). Aquat. Toxicol. 175, 171–183. 10.1016/j.aquatox.2016.03.016 27060237

[B5] Bielen A. Šimatović A. Kosić-Vukšić J. Senta I. Ahel M. Babić S. (2017). Negative environmental impacts of antibiotic-contaminated effluents from pharmaceutical industries. Water Res. 126, 79–87. 10.1016/j.watres.2017.09.019 28923406

[B6] Carvalho J. Lahaye F. Croce J. Schubert M. (2017). CYP26 function is required for the tissue-specific modulation of retinoic acid signaling during amphioxus development. Int. J. Dev. Biol. 61 (10-11-12), 733–747. 10.1387/ijdb.170227ms 29319120

[B7] Christou A. Karaolia P. Hapeshi E. Michael C. Fatta-Kassinos D. (2017). Long-term wastewater irrigation of vegetables in real agricultural systems: concentration of pharmaceuticals in soil, uptake and bioaccumulation in tomato fruits and human health risk assessment. Water Res. 109, 24–34. 10.1016/j.watres.2016.11.033 27865170

[B8] Cui H. Chang H. Zheng H. Wan Y. (2021). Determination and occurrence of sulfonamide transformation products in surface waters. Sci. Total Environ. 779, 146562. 10.1016/j.scitotenv.2021.146562 34030252

[B9] Cui Q. Pan Y. Zhang H. Sheng N. Wang J. Guo Y. (2018). Occurrence and tissue distribution of novel perfluoroether carboxylic and sulfonic acids and legacy per/polyfluoroalkyl substances in black-spotted frog (Pelophylax nigromaculatus). Environ. Sci. Technol. 52 (3), 982–990. 10.1021/acs.est.7b03662 29310433

[B10] Daniel E. Barlow H. R. Sutton G. I. Gu X. Htike Y. Cowdin M. A. (2020). Cyp26b1 is an essential regulator of distal airway epithelial differentiation during lung development. Development 147 (4), dev181560. 10.1242/dev.181560 32001436 PMC7044453

[B11] Dermauw W. Van Leeuwen T. Feyereisen R. (2020). Diversity and evolution of the P450 family in arthropods. Insect Biochem. Mol. Biol. 127, 103490. 10.1016/j.ibmb.2020.103490 33169702

[B12] Emoto Y. Wada H. Okamoto H. Kudo A. Imai Y. (2005). Retinoic acid-metabolizing enzyme Cyp26a1 is essential for determining territories of hindbrain and spinal cord in zebrafish. Dev. Biol. 278 (2), 415–427. 10.1016/j.ydbio.2004.11.023 15680360

[B13] Estabrook R. W. Cooper D. Y. Rosenthal O. (1963). The light reversible carbon monoxide inhibition of the steroid C21-hydroxylase system of the adrenal cortex. Biochem. Z 338, 741–755. 14087340

[B14] Esteves F. Rueff J. Kranendonk M. (2021). The central role of cytochrome P450 in xenobiotic metabolism—a brief review on a fascinating enzyme family. J. Xenobiot. 11, 94–114. 10.3390/jox11030007 34206277 PMC8293344

[B15] Gu X. Xu F. Wang X. Gao X. Zhao Q. (2005). Molecular cloning and expression of a novel CYP26 gene (cyp26d1) during zebrafish early development. Gene Expr. Patterns 5 (6), 733–739. 10.1016/j.modgep.2005.04.005 15979416

[B16] Ha-Duong N. T. Marques-Soares C. Dijols S. Sari M. A. Dansette P. M. Mansuy D. (2001). Interaction of new sulfaphenazole derivatives with human liver cytochrome p450 2Cs: structural determinants required for selective recognition by CYP 2C9 and for inhibition of human CYP 2Cs. Arch. Biochem. Biophys. 394 (2), 189–200. 10.1006/abbi.2001.2511 11594733

[B17] Han J. Won E.-J. Kim H.-S. Nelson D. R. Lee S.-J. Park H. G. (2015). Identification of the full 46 cytochrome P450 (CYP) complement and modulation of CYP expression in response to water-accommodated fractions of crude oil in the cyclopoid copepod paracyclopina nana. Environ. Sci. Technol. 49 (11), 6982–6992. 10.1021/acs.est.5b01244 25942333

[B18] Horton C. Maden M. (1995). Endogenous distribution of retinoids during normal development and teratogenesis in the mouse embryo. Dev. Dyn. 202 (3), 312–323. 10.1002/aja.1002020310 7780180

[B19] Huang F. An Z. Moran M. J. Liu F. (2020). Recognition of typical antibiotic residues in environmental media related to groundwater in China (2009−2019). J. Hazard Mater 399, 122813. 10.1016/j.jhazmat.2020.122813 32937691

[B20] Huang Y. Ding J. Zhang G. Liu S. Zou H. Wang Z. (2021). Interactive effects of microplastics and selected pharmaceuticals on red tilapia: role of microplastic aging. Sci. Total Environ. 752, 142256. 10.1016/j.scitotenv.2020.142256 33207491

[B21] Jiang J. Xie F. Zang C. Cai L. Li C. Wang B. (2016). Assessing the threat status of amphibians in China. Biodivers. Sci. 24, 588–597. 10.17520/biods.2015348

[B22] Kümmerer K. (2009). Antibiotics in the aquatic environment – a review – Part I. Chemosphere 75 (4), 417–434. 10.1016/j.chemosphere.2008.11.086 19185900

[B23] Lampen A. Meyer S. Nau H. (2001). Effects of receptor-selective retinoids on CYP26 gene expression and metabolism of all-trans-retinoic acid in intestinal cells. Drug metabolism Dispos. Biol. fate Chem. 29 (5), 742–747. 11302942

[B24] Lane M. A. Chen A. C. Roman S. D. Derguini F. Gudas L. J. (1999). Removal of LIF (leukemia inhibitory factor) results in increased vitamin A (retinol) metabolism to 4-oxoretinol in embryonic stem cells. Proc. Natl. Acad. Sci. U. S. A. 96 (23), 13524–13529. 10.1073/pnas.96.23.13524 10557354 PMC23981

[B25] Lie K. K. Meier S. Sørhus E. Edvardsen R. B. Karlsen Ø. Olsvik P. A. (2019). Offshore crude oil disrupts retinoid signaling and eye development in larval atlantic haddock. Front. Mar. Sci. 6. 10.3389/fmars.2019.00368

[B26] Lim S. Y. M. Pan Y. Alshagga M. Lim W. Cin K. Alshehade S. A. (2024). CYP14 family in *Caenorhabditis elegans*: mitochondrial function, detoxification, and lifespan. J. Appl. Toxicol. 10.1002/jat.4597 38472099

[B27] Lin H. Liu Z. Yang H. Lu L. Chen R. Zhang X. (2022). Per- and polyfluoroalkyl substances (PFASs) impair lipid metabolism in *Rana nigromaculata*: a field investigation and laboratory study. Environ. Sci. Technol. 56 (18), 13222–13232. 10.1021/acs.est.2c03452 36044002

[B28] Liu X. Lu S. Guo W. Xi B. Wang W. (2018). Antibiotics in the aquatic environments: a review of lakes, China. Sci. Total Environ. 627, 1195–1208. 10.1016/j.scitotenv.2018.01.271 30857084

[B29] Livak K. J. Schmittgen T. D. (2001). Analysis of relative gene expression data using real-time quantitative PCR and the 2(-Delta Delta C(T)) Method. Methods 25 (4), 402–408. 10.1006/meth.2001.1262 11846609

[B30] Maden M. (2002). Retinoid signalling in the development of the central nervous system. Nat. Rev. Neurosci. 3 (11), 843–853. 10.1038/nrn963 12415292

[B31] Manikandan P. Nagini S. (2018). Cytochrome P450 structure, function and clinical significance: a review. Curr. Drug Targets 19 (1), 38–54. 10.2174/1389450118666170125144557 28124606

[B32] Nelson D. R. (1999). A second CYP26 P450 in humans and zebrafish: CYP26B1. Arch. Biochem. Biophys. 371 (2), 345–347. 10.1006/abbi.1999.1438 10545224

[B33] Oliveira L. M. Teixeira F. M. E. Sato M. N. (2018). Impact of retinoic acid on immune cells and inflammatory diseases. Mediat. Inflamm. 2018, 3067126. 10.1155/2018/3067126 PMC6109577 30158832

[B34] Omura T. (1999). Forty years of cytochrome P450. Biochem. Biophys. Res. Commun. 266 (3), 690–698. 10.1006/bbrc.1999.1887 10603307

[B35] Pang L. Shao J. Wen X. Liu D. Zhang Z. Shuang W. (2022). Effect of the neuropathic pain receptor P2X3 on bladder function induced by intraperitoneal injection of cyclophosphamide (CYP) in interstitial cystitis rats. Transl. Androl. 11 (3), 304–312. 10.21037/tau-22-23 PMC8984976 35402189

[B36] Perlmann T. (2002). Retinoid metabolism: a balancing act. Nat. Genet. 31 (1), 7–8. 10.1038/ng877 11953747

[B37] Pikuleva I. A. Cartier N. (2021). Cholesterol hydroxylating cytochrome P450 46A1: from mechanisms of action to clinical applications. Front. Aging Neurosci. 13, 696778. 10.3389/fnagi.2021.696778 34305573 PMC8297829

[B38] Roberts C. (2020). Regulating retinoic acid availability during development and regeneration: the role of the CYP26 enzymes. Int. J. Dev. Biol. 8, 6. 10.3390/jdb8010006 PMC7151129 32151018

[B39] Rutkoski C. F. Grott S. C. Israel N. G. Carneiro F. E. de Campos Guerreiro F. Santos S. (2022). Hepatic and blood alterations in Lithobates catesbeianus tadpoles exposed to sulfamethoxazole and oxytetracycline. Chemosphere 307, 136215. 10.1016/j.chemosphere.2022.136215 36041517

[B40] Rydeen A. B. Waxman J. S. (2014). Cyp26 enzymes are required to balance the cardiac and vascular lineages within the anterior lateral plate mesoderm. Development 141 (8), 1638–1648. 10.1242/dev.105874 24667328 PMC3978838

[B41] Saitou N. Nei M. (1987). The neighbor-joining method: a new method for reconstructing phylogenetic trees. Mol. Biol. Evol. 4 (4), 406–425. 10.1093/oxfordjournals.molbev.a040454 3447015

[B42] Samrani L. M. M. Dumont F. Hallmark N. Bars R. Tinwell H. Pallardy M. (2023). Nervous system development related gene expression regulation in the zebrafish embryo after exposure to valproic acid and retinoic acid: a genome wide approach. Toxicol. Lett. 384, 96–104. 10.1016/j.toxlet.2023.07.005 37451652

[B43] Shao Y. Wang Y. Yuan Y. Xie Y. (2021). A systematic review on antibiotics misuse in livestock and aquaculture and regulation implications in China. Sci. Total Environ. 798, 149205. 10.1016/j.scitotenv.2021.149205 34375247

[B44] Spoorendonk K. M. Peterson-Maduro J. Renn J. r. Trowe T. Kranenbarg S. Winkler C. (2008). Retinoic acid and Cyp26b1 are critical regulators of osteogenesis in the axial skeleton. Development 135 (22), 3765–3774. 10.1242/dev.024034 18927155

[B45] Uehara M. Yashiro K. Takaoka K. Yamamoto M. Hamada H. (2009). Removal of maternal retinoic acid by embryonic CYP26 is required for correct *Nodal* expression during early embryonic patterning. Gene Dev. 23 (14), 1689–1698. 10.1101/gad.1776209 19605690 PMC2714714

[B46] Wang X. Zheng R. Yao Q. Liang Z. Wu M. Wang H. (2019). Effects of fluoride on the histology, lipid metabolism, and bile acid secretion in liver of *Bufo gargarizans* larvae. Environ. Pollut. 254 (Pt B), 113052. 10.1016/j.envpol.2019.113052 31465901

[B47] White J. A. Guo Y. D. Baetz K. Beckett-Jones B. Bonasoro J. Hsu K. E. (1996). Identification of the retinoic acid-inducible all-trans-retinoic acid 4-hydroxylase. J. Biol. Chem. 271 (47), 29922–29927. 10.1074/jbc.271.47.29922 8939936

[B48] White J. A. Ramshaw H. Taimi M. Stangle W. Zhang A. Everingham S. (2000). Identification of the human cytochrome P450, P450RAI-2, which is predominantly expressed in the adult cerebellum and is responsible for all-trans-retinoic acid metabolism. Proc. Natl. Acad. Sci. 97 (12), 6403–6408. 10.1073/pnas.120161397 10823918 PMC18615

[B49] Williams J. A. Kondo N. Okabe T. Takeshita N. Pilchak D. M. Koyama E. (2009). Retinoic acid receptors are required for skeletal growth, matrix homeostasis and growth plate function in postnatal mouse. Dev. Biol. 328 (2), 315–327. 10.1016/j.ydbio.2009.01.031 19389355 PMC4085816

[B50] Wu D. Liu Z. Cai M. Jiao Y. Li Y. Chen Q. (2019). Molecular characterisation of cytochrome P450 enzymes in waterflea (*Daphnia pulex*) and their expression regulation by polystyrene nanoplastics. Aquat. Toxicol. 217, 105350. 10.1016/j.aquatox.2019.105350 31730932

[B51] Xiong J.-Q. Kim S.-J. Kurade M. B. Govindwar S. Abou-Shanab R. A. I. Kim J.-R. (2019). Combined effects of sulfamethazine and sulfamethoxazole on a freshwater microalga, Scenedesmus obliquus: toxicity, biodegradation, and metabolic fate. J. Hazard Mater 370, 138–146. 10.1016/j.jhazmat.2018.07.049 30049519

[B52] Yang J. H. Park J. W. Kim H. S. Lee S. Yerke A. M. Jaiswal Y. S. (2024). Effects of antibiotic residues on fish gut microbiome dysbiosis and mucosal barrier-related pathogen susceptibility in zebrafish experimental model. Antibiot. Basel 13 (1), 82. 10.3390/antibiotics13010082 PMC10812462 38247641

[B53] Zainab S. M. Junaid M. Xu N. Malik R. N. (2020). Antibiotics and antibiotic resistant genes (ARGs) in groundwater: a global review on dissemination, sources, interactions, environmental and human health risks. Water Res. 187, 116455. 10.1016/j.watres.2020.116455 33032106

[B54] Zanger U. M. Schwab M. (2013). Cytochrome P450 enzymes in drug metabolism: regulation of gene expression, enzyme activities, and impact of genetic variation. Pharmacol. Ther. 138 (1), 103–141. 10.1016/j.pharmthera.2012.12.007 23333322

[B55] Zhang D.-D. Wang X.-Y. Chen J.-Y. Kong Z.-Q. Gui Y.-J. Li N.-Y. (2016). Identification and characterization of a pathogenicity-related gene VdCYP1 from Verticillium dahliae. Sci. Rep. 6 (1), 27979. 10.1038/srep27979 27329129 PMC4916405

[B56] Zhang W. Liu Z. Tang S. Li D. Jiang Q. Zhang T. (2020). Transcriptional response provides insights into the effect of chronic polystyrene nanoplastic exposure on *Daphnia pulex* . Chemosphere 238, 124563. 10.1016/j.chemosphere.2019.124563 31454744

