# Supplementary material for: Cytochrome P450 enzymes in the black-spotted frog (Pelophylax nigromaculatus): molecular characterization and upregulation of expression by sulfamethoxazole
Source: Front Physiol. 2024 May 9;15:1412943. doi: 10.3389/fphys.2024.1412943 (PMC11112259; doi:10.3389/fphys.2024.1412943)
Supplement: Supplementary file 1 [file DataSheet1.docx]

**Supplementary material**

**Table S1 Primer sequences, amplicon sizes, and accession numbers of genes used in qPCR.**

| **Genes** | **Forward sequence (5′-3′)** | **Reserve sequence (5′-3′)** | **Amplicon size (bp)** |
| --- | --- | --- | --- |
| *CYP26B1* | CTGACAATGTGCGGAAGATCCTGAT | CCTATGGAGTTGGCGAGACTGTTG | 109 |
| *Actin* | TGGCTTTGGACCTGTTGCTGATG | GCGATGTGAATCTGTCTCTGGACTG | 118 |


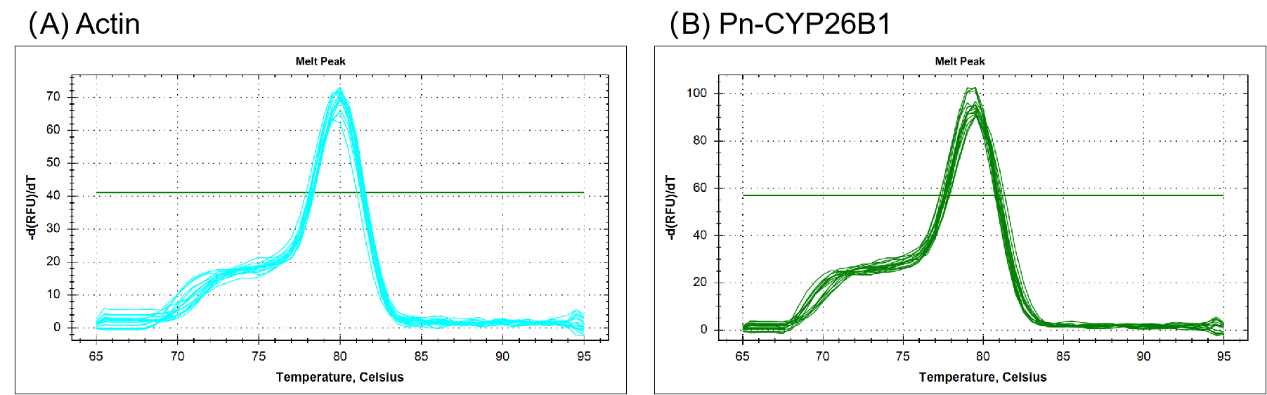


**Figure S1** Melting curves for (A) Actin, (B) Pn-CYP26B1 in *Pelophylax nigromaculatus*.


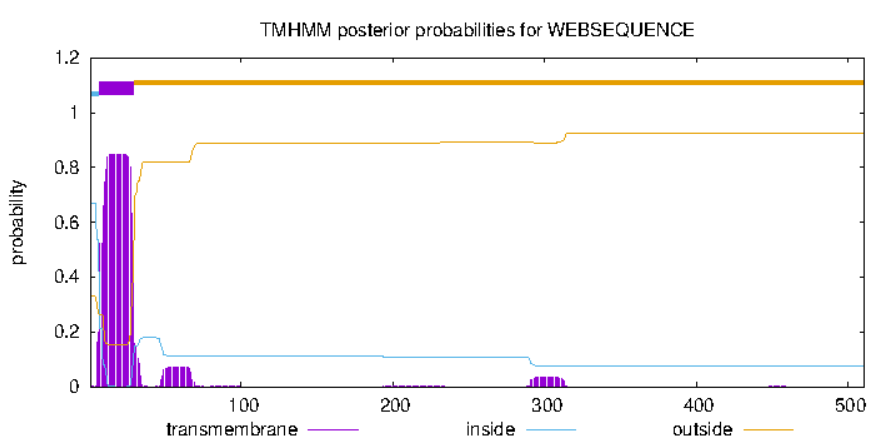


**Figure S2.** Prediction of the transmembrane structural domain of Pn-CYP26B1.
